# Experienced barriers in the use of ICT for social interaction in older adults ageing in place: a qualitative systematic review protocol (SYSR-D-22–00848)

**DOI:** 10.1186/s13643-023-02332-z

**Published:** 2023-10-10

**Authors:** Nina Jøranson, Minna Zechner, Nilufer Korkmaz Yaylagul, Areti Efthymiou, Rosa Silva

**Affiliations:** 1 Faculty of Health Studies, VID Specialized University, Vinderen, PO Box 184, N-3019 Oslo, Norway; 2 University of Helsinki, Faculty of Social Sciences, University of Helsinki, Helsinki, P.O. Box 54 (Unioninkatu 37), 00014 Finland; 3 Faculty of Health Science, Gerontology Department, Akdeniz University, Dumlupınar Boulevard, 07058 Antalya, Turkey; 4 Quality of Life Lab, Department of Social Work, Hellenic Mediterranean University, Estavromenos Heraklion, Crete PC 71410 Greece; 5 Center for Health Technology and Services Research (CINTESIS), Nursing School of Porto, 4200-072 Porto, Portugal; 6 Portugal Centre for Evidence Based Practice, A JBI Centre of Excellence (PCEBP), 3000-232 Coimbra, Portugal

**Keywords:** Older adults, Social interaction, Social networks, Ageing in place, Information and communication technology, Loneliness, Social isolation, Barriers

## Abstract

**Background:**

Several information and communication technologies (ICT) have been developed to enhance social connectedness of older adults aging in place, although they are not accessible for all. Barriers in using ICT might for example relate to health issues, sensory loss, lack of technical skills, or reluctance to use technologies. Though knowledge on these barriers is crucial for the development and implementation of ICT for older adults, no systematic review was found on this subject. The proposed qualitative systematic review aims to explore barriers experienced by older adults aging in place in using ICT for social interaction.

**Methods:**

The review will be conducted in accordance with the JBI methodology. Databases for search will include MEDLINE (via PubMed), CINAHL (via EBSCO), and Web of Science (ISI), among others. Included studies focus on older adults ageing in place 60 years or older. Pairs of authors will independently, by following agreed guidelines, assess the eligibility of studies, and extract data. The testing of eligibility criteria and screening of titles, abstracts, and full texts will be performed. The findings will describe for example populations, context, culture, and the phenomena of interest. Qualitative research findings will, where possible, be pooled by using JBI SUMARI for the meta-aggregation approach.

**Discussion:**

The mapping of published studies has the potential to identify research gaps in the existing literature, which again may inform developers and stakeholders in designing more user-friendly and adaptive ICT solutions for older people ageing in place.

**Systematic review registration:**

CRD42022370044.

**Supplementary Information:**

The online version contains supplementary material available at 10.1186/s13643-023-02332-z.

## Introduction

Since the 1970s, the term *ageing in place* has become one of the buzzwords of policies and research addressing policies of care in the context of ongoing demographic transition [1]. The Center for Disease Control defines ageing in place as the capacity for individuals to securely and independently live in their own home and neighborhoods, regardless of economic status, ability, or age, with the goal of staying at home for as long as possible, using minimal care services, and maintaining independence in the community [2].

Thus, ageing in place emphasizes the desire for secure and independent living in familiar surroundings, regardless of age, resources or ability. Minimizing reliance on care services and nursing homes while preserving community connections is key to achieving this goal [3–5]. In this context, Information and Communication Technologies (ICT) hold a promise as a solution to enhance social interaction and reduce loneliness, which are crucial factors in promoting successful aging in place. However, addressing barriers to ICT adoption among older adults is essential to maximize the positive impact of these technologies and to empower older adults to successfully age in place.

Reviews have explored many aspects of supporting older adults to maintain aging in place. These included various sites of aging, different forms of support, personal characteristics of older adults, technology, and social networks [1, 6].

The role of social networks and social connectedness in individual health and well-being is significant. The lack and limited amount of social interaction might cause loneliness and social isolation [7]. Holt-Lunstad and colleagues [8] define loneliness as a subjective feeling of being alone, and social isolation to be an objective lack of social connections. Social connections also impact possibilities to receive care services, because people with more extensive social networks have better opportunities to receive not only family but also home care later in life [9]. Social networks tend to shrink by age, as contemporaries become frail and decease. This is essential particularly in Northern Europe, where cohabitation with adult children is uncommon [10–13]. Loneliness and social isolation are severe problems faced by many older adults who age in place, in particular for those who experience barriers in physical mobility [11, 14–16]. Various technological applications for social interaction have been offered as one of the solutions to alleviate loneliness and promote greater well-being in aging populations [17]. These information and communication technologies (ICT) are systems created by integrated use of computer and communication technologies [6, 18] that enable older adults to be informed and to establish, form or maintain mutual relationships with people and society [19]. Some of these technological applications include social media platforms, video conferencing and video calls, online communities, and forums [6, 17, 18], that leverage the power of digital connectivity to address social isolation and promote social interactions. However, some older adults experience the positive aspects of solitude, which means that being alone is not a problem for everybody [20].

Several types of ICT have been developed to alleviate social isolation among older adults. Khosravi and colleagues [21] reviewed empirical studies on these ICTs and their effectiveness in alleviating social isolation. They divided the various ICT solutions into six categories, and their assessment shows some potential for reduced loneliness and social isolation. Most included studies focused on general ICTs, such as Internet, e-mail, computers, tablet pads, and smartphones at large. These devices, programs and technologies provide multiple ways for older adults to communicate with family and friends. Other ICTs included social network sites, such as Twitter and Facebook, which assist older adults to build and continue social relations overcoming both mobility problems and geographical distances. The third group of loneliness-reducing technologies consisted of robots, that mainly provide a sense of social presence and the possibility for communication. The fourth group included videos that offer physical and cognitive stimulation as well as mimic real life social interaction. The fifth group involved software that support social connectivity, memory, and leisure activities by acting as a reminder and a kind of companion as well. Other types of relevant technologies were Tele-Care solutions that provide monitoring, communication, and support for older adults as well as 3D virtual environments [21].

There are several studies that show various types of ICT or social interaction technologies to have positive impact on social connectedness and to decrease social isolation and loneliness in older adults aging in place [10, 22–24]. These ICTs have the potential to be useful for current and future societies. After all, many older adults are familiar with such. According to Eurostat [25] 61% of those aged 65–74 in the European Union, used the internet during the last three months of 2020. However, for many older adults ICT is not accessible, available, or desirable. Older adults appear to be the population facing most difficulties and are less confident in using new technology compared with the younger ones [26, 27]. Older users do not necessarily take advantage of the services available in the Internet, and there are large numbers of older adults who do not search for health information or maintain social networks online [27]. Additionally, several statistics such as Eurostat, do not cover the oldest age groups, such as 75 and older. This is the group of older adults who are most likely to face mobility barriers that affect social interaction which then could be overcome by ICT.

There are several challenges that hinder older adults’ use of ICT. These include difficulties in having the appropriate technology or necessary resources to set up the devices and services. Problems with health, hearing or vision, lack of skills as well as computer anxiety may also limit the use of ICT [21, 28, 29]. Zaman and colleagues [30] reported barriers of ICT use in older adults with chronic diseases caused by knowledge gaps, unwillingness to adopt new skills, and reluctance to use technological solutions. In addition, many older adults face digital outsiderness or disengagement, and the use of the Internet and ICT tend to decrease with age due to deteriorating health, living in the margins, or facing psychological issues [31]. A systematic literature review by Yap et al. [32] identified seven factors of antecedents, which influence older adults’ technology adoption. These contain technological factors related to usefulness, psychological factors about attitudes and anxiety towards ICT usage, social factors containing social pressure and support, personal factors such as age and sensory difficulties, costs, environment that can facilitate or hinder technology use, and finally behavioral factors that connect with the habits and frequency of use.

Social interaction technologies have several characteristics that could facilitate social connectivity through real-time communication, user-generated content, and social media platforms. Other characteristics are prioritizing privacy and mobile accessibility, employing gamification and data analytics, and incorporating Virtual and Augmented Reality for immersive experiences, as well as integration with other services and emphasis on visual content enhance engagement in this ever-evolving field. However, these characteristics may, in certain circumstances, also be barriers for older users.

It is important to assess the barriers for using ICT as it helps to develop more suitable technologies for those who are able and willing to use them. Recognizing the wide range of ICTs, this review focuses on social interaction technologies, as it is understood that they can be tools to strengthen social interaction. Hence, conducting a qualitative systematic review on the barriers faced by older adults aging in place when using ICT to strengthen interaction is essential to identifying effective strategies for promoting successful aging in place. A preliminary search in several databases identified no similar qualitative systematic reviews or protocols besides a scoping review protocol on technological interventions for reducing loneliness [33]. The present systematic qualitative review focuses on barriers that older adults aging in place face when using ICT to strengthen interaction and reduce loneliness.

## Methods

The protocol is registered in the International Prospective Register for Systematic Reviews (PROSPERO CRD42022370044). The systematic review will be conducted in accordance with JBI methodology for systematic reviews of qualitative evidence [34].

### Study objectives

The objective of this systematic review is to explore published studies describing how older adults aging in place have used ICT (information and communication technology) for social interaction at home to analyse and systematize their experienced barriers in regards such technologies. The research question to answer the study objectives is *What are the barriers experienced by older adults ageing in place in using ICT for social interaction?*

Specific objectives of the review to answer the research question are:

- Identify the characteristics of social interaction technologies that have produced experienced barriers for older adults.
- Explore the barriers *experienced by* older adults in the use of social interaction technologies.

### Inclusion and exclusion criteria

The inclusion and exclusion criteria of the identified studies are described in Table 1.

**Table 1:** Inclusion and exclusion criteria

Criteria	Inclusion	Exclusion
Participants	Older adults 60 years or older of any level of functional status (with or without disability)	
Phenomena of interest	Explore barriers towards using communication technologies for social interaction, that could have of the different characteristics such as connectivity and communication, social networking, user-generated content, social feedback and validation, real-time updates, groups, and communities The phases of the technology adoption processes will be considered, specifically the Rogers' innovation diffusion model [35]	Studies where participants use communication technologies for other purposes than social interaction or for social interaction and simultaneously for other purposes such as physical exercise or telemedicine
Context	Older adults ageing in place: at home/community-dwelling/supported housing of any geographical location. Ageing in place means individuals can live independently (may receive assistance) and securely in their homes and communities, regardless of age, ability, or economic status. The goal is to stay at home with minimal care services and maintain sufficient independence [2]	Older adults residing in nursing homes or institutions
Type of study	Published in peer reviewed scientific journals	Master and PhD thesis, lecture notes, other reviews and meta-analysis, books, book chapters, comments, conference proceedings and ongoing studies (e.g., protocols)
Methods and study design	Qualitative studies and mixed method	Studies not responding to at least 5 out of 10 assessment items in the standard JBI critical appraisal checklist for qualitative research [34] For more details, please see the assessment of methodological quality section
Language	English, Finnish, Greek, Portuguese, Spanish, the Scandinavian languages, and Turkish	

### Types of studies

This review will assess peer-reviewed qualitative studies including, but not limited to, methodologies and designs related to phenomenology, hermeneutics, grounded theory, ethnography, and action research. Qualitative results from mixed method studies will also be included.

### Search strategy

The search strategy aims to locate published and peer-reviewed studies. An initial limited search of CINAHL (via EBSCO, see Appendix 1) was undertaken to identify articles on the topic. The text words contained in the titles and abstracts of relevant articles, and the index terms used to describe the articles will be used to develop a full search strategy for CINAHL via EBSCO.

Initial keywords in different forms to be used will be “elder”, “old’’, “senior”, “aged”, “home residing”, “independent living”, “living community”, “community dwelling”, “home environment”, “home-based”, “communication technology”, “digital communication”, “ICT”, “information and communication technology”, “connectivity”, “real-time communication”, “user-generated content”, “collaboration tools”, “virtual and augmented reality, “experiences”, “barrier”, “obstacle”, “non-use”, “non-take-up”, “difficult”, “social inclusion”, “social isolation”, “social interaction”, “social networking”, “loneliness”.

The search strategy, including all identified keywords and index terms, will be adapted for each included information source. The references of all studies selected for critical appraisal will be screened for additional data.

The databases to be searched include Academic Search Complete, CINAHL (EBSCO), Cochrane CENTRAL, ERIC (Education Resources Information Center), MedicLatina, MEDLINE (PubMed), ProQuest, Psychology and Behavioral Sciences Collection, SciELO (Scientific Electronic Library Online), Science Direct, Social Science Citation Index, Scopus, Web of science (ISI) and IEEE Xplore.

### Study selection

Following the search, all identified citations will be collated and uploaded into Mendeley (Mendeley Ltd., Elsevier, Netherlands) and duplicates will be removed. Titles and abstracts will then be screened by two independent reviewers using Rayyan–Intelligent Systematic Review [36], for assessment against the inclusion criteria. Potentially relevant studies will be retrieved in full, and their citation details imported into the JBI System for the Unified Management, Assessment and Review of Information (JBI SUMARI; Adelaide, Australia) [37].

The selected full texts will be assessed in detail towards the inclusion criteria by pairs of reviewers. The grounds for excluding full texts will be recorded and reported in the systematic review. Any disagreements that arise between the reviewers at any stage of the selection process will be resolved through discussion or by engaging a third reviewer. The results of the search will be reported in full in the final systematic review and will be presented in the Preferred Reporting Items for Systematic Reviews and Meta-analyses (PRISMA) flow diagram [38].

### Assessment of methodological quality

All eligible studies will be critically appraised by reviewer pairs to assess methodological quality by using the standard JBI critical appraisal checklist for qualitative research [34] before undergoing data extraction and synthesis. The questions in the checklist relate to consistency in methodology, philosophical perspective, research question or objectives, data collection, data representation and analysis, representation of results, researcher position, participant voices, ethical issues, and the quality of reporting. The list helps to ensure the quality of included studies and identify possible biases. Any disagreements between the reviewers during the assessment process will be resolved through discussion, or by engaging a third reviewer. The results of critical appraisal will be reported in textual and table forms.

After critical appraisal, studies that do not meet a predetermined threshold of quality will be excluded. Studies that receive a classification of “no” or “unclear” for five or more out of the 10 questions (50% or more) in the critical appraisal checklist will be excluded. Studies classified as “no” or “uncertain” for one to five questions will be discussed in a meeting where two reviewers will determine whether these studies will be included. Any disagreements arising between the reviewers will be resolved through discussion or with a third reviewer. Authors of the articles will be contacted to request missing or additional data for clarification when necessary. The results of the critical evaluation will be presented in a narrative format and in a table.

In mixed studies, ensuring the methodological quality of the included research is crucial to capture participants’ voices and meanings effectively. To achieve this, the JBI critical appraisal checklist for qualitative research will be applied. Only studies that answer “yes” to the following questions will be considered for inclusion: Q2 (Is there alignment between the research methodology and research questions or objectives?), Q3 (Is there alignment between the research methodology and data collection methods?), Q4 (Is there alignment between the research methodology and data representation and analysis?), and Q8 (Are participants and their voices adequately represented?) [34].

### Data extraction

Qualitative data will be extracted from papers included in the review by two independent reviewers using the standardized data extraction tool in JBI SUMARI [37]. The data extracted will include specific details about the populations, context, culture, geographical location, study methods, and the phenomena of interest relevant to the review questions. The findings and their illustrations will be extracted and assigned a level of credibility. Any disagreements that arise between the reviewers during data extraction will be resolved through discussion or by engaging a third reviewer. Authors of papers will be contacted to request missing or additional data, where required.

If at this point missing data is detected (i.e. on assessment of methodological quality or data extraction), authors of selected papers will be contacted to request missing or additional data.

### Data synthesis

Qualitative research findings will be, where possible, pooled using JBI SUMARI with the meta-aggregation approach [37]. This involves the aggregation or synthesis of findings to generate a set of statements that represent that aggregation, through assembling the findings, and categorizing these findings based on similarity in meaning, based on the type of technology and the type of subgroup of participants involved (healthy, unhealthy and disabled, and the latter ones will be subcategorized according to the illnesses or disabilities). These categories will then be subjected to a synthesis to produce a single comprehensive set of synthesized findings that can be used for evidence-based practice, to guide future research or impact the user-friendliness in development of ICT products for social interaction, and according to the type or characteristics of the group/subgroup of participants (healthy/unhealthy/disabled). Where textual grouping is not possible, the findings will be presented in textual format. Only unequivocal and credible findings are included in the synthesis [34].

### Assessing confidence in the findings

The final synthesized findings will be graded according to the ConQual (confidence in qualitative synthesis findings) approach for establishing confidence in the output of qualitative research synthesis and will be presented in a Summary of Findings [39]. The ConQual approach offers a means of grading the credibility and dependability of the findings of a review [39].

Dependability will be determined for each study by analyzing methodological quality appraisal (questions 1–5 of the JBI critical appraisal checklist for qualitative research) [34]. Credibility will be assessed by the quality and evident detail of illustrations of findings by measures of unequivocal (U), credible (C), or not supported (NS) [34].

Each synthesized finding from the review will be presented, along with the type of research informing it, and scored for dependability and credibility, and given an overall ConQual score. The authors of the review will be consulted and confirmed the appraisal, extraction, and synthesis of findings. Thus, the Summary of Findings will include the major elements of the review and details on the determined ConQual score.

Included in the Summary of Findings will be the title, population, phenomena of interest, and context for the specific review. Each synthesized findings from the review will then be presented, along with the type of research informing it, with scores for dependability and credibility, and the overall ConQual score [39].

## Discussion

Here the rationale and the design of the qualitative systematic review has been introduced to answer the research question “What are the barriers experienced by older adults ageing in place in using ICT for social interaction?” Several studies describe ICT as useful for social engagement for older adults ageing in place [10, 22–24]. Thus, this review can help to shed light on potential solutions to enhance the development of age-appropriate technologies and the use of ICT among older adults and support their well-being, independence, and social connectedness as they age in place. By fostering the effective integration of easy-to use and appropriate ICT in the lives of older adults willing to use these technologies, we can create a future where aging in place is at least socially fulfilling and enriching experience for all older adults.

Several and various technologies have been developed for socializing purposes [21, 22]. However, the literature also points out that ICT is not accessible for all, and several barriers, such as technical issues, deteriorating health, sensory loss, and lack of technical skills are experienced by older adults [21, 28, 29]. In addition, many studies describe experiences of digital disengagement towards technical devices or ICT in older adults [31, 32], which makes implementation and use of ICT difficult. This review will provide a systematic overview of barriers that older adults experience in relation to ICT-use for social interaction that is presented in this growing research field. In addition, the meta-aggregation approach will generate a comprehensive set of synthesized findings. The review will identify gaps in the existing literature to inform policy-makers, ICT developers, and other stakeholders in designing more user-friendly and adaptive ICT solutions for older adults aging in place.

### Supplementary Information

**Additional file 1: Appendix I.** Search strategy.

## Data Availability

Not applicable.

## Abbreviations

ICT: Information and communication technologies
JBI: Joanna Briggs Institute
SUMARI: System for the Unified Management, Assessment and Review of Information
PRISMA: Preferred Reporting Items for Systematic Reviews and Meta-analyses
ConQual: Confidence in qualitative synthesis findings
