# Supplementary material for: Experienced barriers in the use of ICT for social interaction in older adults ageing in place: a qualitative systematic review protocol (SYSR-D-22–00848)
Source: Syst Rev. 2023 Oct 10;12:192. doi: 10.1186/s13643-023-02332-z (PMC10563227; doi:10.1186/s13643-023-02332-z)
Supplement: Supplementary file 1 — Additional file 1: Appendix I. Search strategy. [file 13643_2023_2332_MOESM1_ESM.docx]

**Appendix I: Search strategy**

CINAHL (via EBSCO)

Search conducted: August 9th, 2023

| Search ID # | Search strategy | Record |
| --- | --- | --- |
| S13 | S3 AND S11 AND S12 | 1,483 |
| S12 | TI (“Home residing” OR “Living Community” OR “Community dwelling” OR “Community-dwelling” OR “Home environment” OR “ageing in place” OR “age in place” OR “aging in plaque” or “living at home”) OR AB (“Home residing” OR “Living Community” OR “Community dwelling” OR “Community-dwelling” OR “Home environment” OR “ageing in place” OR “age in place” OR “aging in place” OR “living at home”) | 26,413 |
| S11 | S6 AND S10 | 246,108 |
| S10 | S7 OR S8 OR S9 | 1,509,633 |
| S9 | (MH "Communications Media+") | 697,892 |
| S8 | (MH "Communication+") | 326,732 |
| S7 | TI (Technolog* OR digital* OR Telepresence* OR ICT* OR “Tele-Care solution” OR “Tele-Care solutions” OR connectivit* OR “user-generated content” OR “user-generated contents” OR “collaboration tools” OR “collaboration tool” OR virtual* OR “augmented reality” OR “augmented realities” OR “social interaction technology” or “social interaction ICT” or Communication* or Information*)  OR AB (Technolog* OR digital* OR Telepresence* OR ICT* OR “Tele-Care solution” OR “Tele-Care solutions” OR connectivit* OR “user-generated content” OR “user-generated contents” OR “collaboration tools” OR “collaboration tool” OR virtual* OR “augmented reality” OR “augmented realities” OR “social interaction technology” or “social interaction ICT” or Communication* or Information*) | 765,516 |
| S6 | S4 OR S5 | 769,840 |
| S5 | (MH "Communication Barriers+") | 6,152 |
| S4 | TI (Barrier* OR experience* OR Obstacle* OR Non-use OR Non-take-up OR E-exclusion OR Dropout OR Withdraw* OR outsiderness OR disengagement* OR perception*) OR AB (Barrier* OR experience* or Obstacle* OR Non-use or Non-take-up OR E-exclusion or Dropout or Withdraw* OR outsiderness OR disengagement* OR perception*) | 766,724 |
| S3 | S1 OR S2 | 1,891,828 |
| S2 | (MH "Aged+") | 949,949 |
| S1 | TI (Elder* OR “Older adult” OR “older adults” OR “Older person” OR 60+ OR Senior* OR “Older individual” OR “Older individuals” OR Age* or Aging) OR AB (Elder* OR “Older adult” OR “older adults” OR “Older person” OR 60+ or Senior* OR “Older individual” OR “Older individuals” OR Age* OR Aging) | 1,345,978 |
